# Anti-CGRP Monoclonal Antibodies: the Next Era of Migraine Prevention?

**DOI:** 10.1007/s11940-017-0463-4

**Published:** 2017-06-27

**Authors:** Amy R. Tso, Peter J. Goadsby

**Affiliations:** 1 Basic and Clinical Neuroscience, Institute of Psychiatry, Psychology and Neuroscience, King’s College London, London, UK; 2 NIHR—Wellcome Trust King’s Clinical Research Facility, King’s College Hospital, Wellcome Foundation Building, London, SE5 9PJ UK

**Keywords:** Migraine, Preventive, Treatment, Antibody, CGRP

## Abstract

Migraine is a very disabling disorder with severe impact on patients’ lives and substantive costs to society in terms of healthcare costs and lost productivity. Prevention is a key component of migraine therapy, and while numerous preventive options exist, each is burdened by either troublesome side effects or insufficient efficacy. All migraine preventives currently in clinical use were licensed for other purposes and, by chance, have efficacy against migraine. As our understanding of migraine has evolved, calcitonin gene-related peptide (CGRP) has moved to the forefront as a neuropeptide central to migraine pathophysiology. Six small molecule CGRP receptor antagonists were shown to be effective for acute treatment of migraine; two were stopped for hepatotoxicity or one for formulation concern issues and one is now in phase III. Monoclonal antibodies against CGRP or the CGRP receptor have a longer duration of action and have been investigated for migraine prevention. Four are in development and three have completed phase II and one phase III trials; every reported study has been positive. Furthermore, no safety issues have arisen to date, including hepatic or cardiovascular effects, and initial tolerability appears to be excellent. Monoclonal antibodies antagonizing the CGRP pathway represent a novel approach to prevention: a mechanism-specific migraine-targeted therapy. While we must await the results of all the phase III trials, cautious excitement seems warranted as we enter a new era of better tolerated, well-understood, bespoke migraine treatment for this common and disabling neurological disorder.

## Introduction

Migraine is one of the most prevalent and disabling disorders in the world, yet its pathophysiology remains incompletely understood [1]. Perhaps relating to this, no preventive treatments that are currently in clinical use were developed specifically for migraine [2]. For acute treatment, the only class of agents developed specifically for migraine, the triptans, was in fact thought to act via its vasoconstrictive properties [3] based on the prevailing view at the time that migraine was a vascular disorder [4]. It is now clear that vasodilation is neither necessary nor sufficient for migraine pain, and migraine is now understood to be a complex neuronal disorder with vascular epiphenomenon [5••].

The need for improved preventive treatments for migraine is apparent: in the 2015 Global Burden of Disease Study, migraine was the seventh leading cause of disability globally and the leading neurological cause of disability, accounting for over half of the years lost to disability from all neurological disorders [6]. In the USA, the estimated direct healthcare expenditure on migraine is approximately $9 billion per year [7] with indirect costs due to lost productivity doubling that figure [8]. Although numerous preventive treatment options exist [2], their utility is often limited by intolerable side effects such as cognitive slowing, drowsiness, or weight gain In general, patient adherence is low due to such side effects or insufficient efficacy [9, 10]

Research is ongoing to address this unmet need for better prevention with fewer side effects. Advances in our understanding of migraine pathophysiology have led to new approaches such as neuromodulation and new pharmacologic targets [11, 12], and perhaps none have been as promising as calcitonin gene-related peptide (CGRP).

## Calcitonin gene-related peptide

CGRP is a 37-amino acid neuropeptide that is widely distributed throughout the central and peripheral nervous systems [13, 14]. CGRP is present in sensory neurons, including in the trigeminal ganglion and nerve endings as well as dorsal root ganglia [15••]. CGRP has been localized in unmyelinated C fibers and small myelinated Aδ fibers, which are involved in pain transmission, and is co-expressed with serotonin 5-HT_1B_ and 5-HT_1D_ receptors [16, 17]. Immunohistochemistry studies in rats have shown CGRP is present centrally in brain structures including the hypothalamus, thalamus, and cerebellum [18], and in humans, CGRP binds densely in the cerebellum [19].

CGRP exists in α and β isoforms. The main isoform expressed in the trigeminovascular system and in the brain is αCGRP, which is formed from alternative splicing of the calcitonin gene. The β isoform is transcribed from a different gene and differs from αCGRP by three amino acids. It is expressed primarily in the enteric nervous system where it has distinct physiologic functions such as inhibiting gastric acid secretion [18].

CGRP binds to a G-protein coupled receptor formed by two subunits: calcitonin receptor-like receptor (CLR) and receptor activity-modifying protein 1 (RAMP1) [20]. Outside of the nervous system, the CGRP receptor is also found throughout the arterial system in the smooth muscle cell layer, including the cardiovascular and cerebrovascular systems, as well as in the adrenal glands, kidneys, and pancreas [16, 21].

In addition to being a potent vasodilator [22], evidence suggests that CGRP plays an important role in migraine pathophysiology. During spontaneous migraine attacks, CGRP concentrations measured from the external jugular vein rise [23] and CGRP serum levels decrease after administration of triptans in parallel with symptomatic relief [24, 25]. Serum CGRP levels are elevated interictally in chronic migraine and to a lesser extent in episodic migraine [26]. Lastly, intravenous infusion of CGRP triggers attacks in migraineurs that are indistinguishable from spontaneous attacks [27, 28].

Antagonism of the CGRP pathway has been pursued as a new strategy for both acute and preventive treatment of migraine with promising results [12].

## Clinical trials

The CGRP mechanism can be antagonized by targeting either the peptide or its receptor with either small molecule antagonists or monoclonal antibodies. This was first attempted for acute treatment of migraine. Several antagonists of the CGRP receptor including olcegepant (BIBN 4096 BS), telcagepant (MK-0974), MK-3207, BI 44370 TA, and BMS-927711 resulted in significantly higher pain-free rates at 2 h when compared to placebo [29–33]. Unfortunately, development was complicated by pharmacokinetic issues and for telcagepant and MK-3207 by hepatotoxicity. Ubrogepant is still under development for acute treatment [34] and atogepant for prevention (NCT02848326). Despite initial setbacks, these clinical trials have provided the proof of principle that targeting the CGRP pathway can effectively treat migraine.

Telcagepant was also investigated for prevention of episodic migraine [35]. Subjects with 3 to 14 days of headache during a 4-week baseline period were randomized to receive a daily dose of telcagepant 140 or 280 mg or placebo for 12 weeks. The trial was terminated prematurely due to hepatotoxicity concerns; 13 patients in the telcagepant group and none in the placebo arm had derangement of liver function tests greater than three times the upper limit of normal. At the time of termination, all patients (*n* = 660) had been randomized, most had completed 1 month of treatment (*n* = 593), and some had completed 2 months of treatment (*n* = 312). Efficacy analysis could not be performed as originally planned, but the data suggested a reduction in the primary efficacy endpoint, mean monthly headache days, in the telcagepant groups compared to placebo after 1 month.

Monoclonal antibodies have been used increasingly for treatment of neurological disorders [36, 37], and migraine has been no exception. Monoclonal antibodies have a long half-life that makes them suitable for therapies requiring chronic activity such as migraine prevention. Furthermore, their long duration of action allows for less frequent dosing, e.g., once or twice monthly. Finally, antibodies are highly specific, allowing for highly selective targeting of either CGRP or its receptor.

Four monoclonal antibodies are currently in development for migraine prevention: three against CGRP itself: galcanezumab (LY2951742), eptinezumab (ALD403), and fremanezumab (TEV-48215) and one against the CGRP receptor erenumab (AMG-334). Results from phase II trials have been published for all four compounds (Table 1), and phase III trials are underway or completed. Phase III results have not been published to date.

**Table 1:** Summary of anti-CGRP antibody phase II trials

	Study population	Dose	Primary endpoint	Time	Results
Eptinezumab (ALD403)	18–55 years, episodic migraine (5–14 days/month)	1000 mg IV, single dose	Change in migraine days/4 weeks	Week 5–8	Active (*n* = 81) - 5.6 days, placebo (*n* = 82) - 4.6 days *p* = 0.03 (one-sided)
Galcanezumab (LY2951742)	18–65 years, episodic migraine (4–14 days/month)	150 mg SC q2wk for 12 wk	Change in migraine days/4 weeks	Week 9–12	Active (*n* = 108) - 4.2 days, placebo (*n* = 110) - 3.0 days *p* = 0.003
Fremanezumab (TEV-48215 or LBR-101)	18–65 years, high-frequency episodic migraine (8–14 days/month)	225 mg SC q4wk for 12 weeks	change in migraine days/4 weeks	Week 9–12	Active (*n* = 95) - 6.27 days, placebo (*n* = 104) - 3.46 days *p* < 0.0001
		675 mg SC q4wk for 12 weeks			Active (*n* = 95) - 6.09 d, placebo (*n* = 104) - 3.46 d *p* < 0.0001
	18–65 years, chronic migraine (15+ days/month)	675 mg/225 mg/225 mg SC q4wk	Change in headache hours of any severity/4 weeks	Week 9–12	Active (*n* = 87) - 59.8 h, placebo (*n* = 104) - 37.1 h *p* = 0.0386
		900 mg SC q4wk for 12 weeks			Active (*n* = 86) - 67.5 h, placebo (*n* = 104) - 37.1 h *p* = 0.0057
Erenumab (AMG-334)	18–60 years, episodic migraine (4–14 days/mo)	7 mg q4wk for 12 weeks	change in migraine days/4 weeks	Week 9–12	Active (*n* = 108) - 2.2 days^a^
		21 mg q4wk for 12 weeks			Active (*n* = 108) - 2.4 days^a^
		70 mg q4wk for 12 weeks			Active (*n* = 107) - 3.4 days, placebo (*n* = 160) - 2.3 days *p* = 0.021

^a^not statistically significantly different from placebo

*Galcanezumab* (LY2951742): The first phase II clinical trial results to be published were for Eli-Lilly’s galcanezumab [38]. This study randomized patients with episodic migraine (4 to 14 headache days in 4-week baseline period) to galcanezumab 150 mg subcutaneously versus placebo every 2 weeks for 12 weeks. Primary efficacy endpoint was the change in number of migraine days during the third 4-week treatment period (weeks 9–12) compared to the baseline period. The mean change in migraine headache days was significantly different in the galcanezumab group compared to the placebo group (−4.2 versus −3.0 days, respectively; least squares mean difference 1.2, *p* = 0.0030).

*Eptinezumab* (*ALD-403*): Alder Biopharmaceuticals took a slightly different approach with eptinezumab, reasoning that intravenous administration would result in rapidly efficacious dosing with immediate physiological effect. Patients with episodic migraine (5 to 14 headache days in 4-week baseline period) were randomized to either a single dose of monthly intravenous eptinezumab 1000 mg or placebo. The primary efficacy endpoint was the change in number of migraine days during weeks 5–8 compared to the baseline period. With a one-sided *p* value (pre-specified), eptinezumab resulted in significantly fewer migraine days compared to placebo (−5.6 versus −4.6 days, respectively; difference 1.0, *p* = 0.0306) [39].

*fremanezumab* (TEV-48215 or LBR-101): Teva Pharmaceuticals investigated fremanezumab in two separate trials for patients with either high-frequency episodic migraine or chronic migraine [40, 41]. Patients with 8 to 14 headache days in 4-week baseline period were randomized to subcutaneous injections of either fremanezumab 225 or 675 mg or placebo every 4 weeks for 12 weeks. Primary efficacy endpoint was the change in number of migraine days during the third 4-week treatment period (weeks 9–12) compared to the baseline period. The least square mean reduction in migraine days was significantly greater compared to placebo for both the fremanezumab 225 mg (−6.27 versus −3.46 days; difference 2.81 days, *p* < 0.0001) and 675 mg doses (−6.09 versus −3.46 days; difference 2.64 days, *p* < 0.0001). Of note, in contrast to the previous two studies, patients were not excluded for use of a migraine preventive; use of one preventive was allowed provided the dose had been stable for 2 months prior to screening.

For the chronic migraine trial, patients were randomized to either placebo or one of two fremanezumab doses given every 4 weeks for 12 weeks: 675 mg loading dose followed by two 225 mg doses or three doses of 900 mg. Primary efficacy endpoint was change in hours of headache of any severity during the third 4-week treatment period (weeks 9–12) compared to the baseline period. The least square mean reduction in headache hours of any severity was significantly greater compared to placebo for both the fremanezumab 675 mg/225 mg /225 mg (−59.8 versus −37.1 h; difference 22.7 h, *p* = 0.0386) and 900 mg doses (−67.5 versus −37.1 h; difference 30.4 h, *p* = 0.0057). Use of up to two preventives was permitted provided the doses were stable for at least 3 months. Of note, although chronic migraine is defined as 15 or more days of headache per month, the mean number of headache days per month in the study population was approximately 16. Thus, the results may not be generalizable to patients with daily or near daily headache.

*Erenumab* (AMG-334): Lastly, Amgen has developed a monoclonal antibody against the CGRP receptor, erenumab, in contrast to the other three antibodies that are targeted at the CGRP molecule itself. Patients with episodic migraine (4 to 14 headache days in 4-week baseline period) were randomized to either placebo or one of three doses of erenumab (7, 14, or 70 mg) subcutaneously every 4 weeks for 12 weeks. Primary efficacy endpoint was the change in number of migraine days during the third 4-week treatment period (weeks 9–12) compared to the baseline period. The least square mean change in migraine headache days was significantly different from the placebo group only for the highest dose, erenumab 70 mg (−3.4 versus −2.3 days; difference 1.1 days, *p* = 0.021) [42].

## Safety and tolerability

Initial safety and tolerability data from phase II trials appears excellent for the anti-CGRP monoclonal antibodies. No clinically significant change in vitals or ECGs was observed. Importantly, no change in hepatic enzymes judged to be treatment-related was seen with any of the monoclonal antibodies, in contrast to the small molecule CGRP receptor antagonists telcagepant and MK-3207. Adverse events were reported in a similar proportion of patients in the placebo and treatment groups. The most common treatment-related adverse event was injection site reaction of mild to moderate severity with subcutaneous injections; no intravenous infusion reactions were seen with eptinezumab. No treatment-related serious adverse events were reported.

Although all the monoclonal antibodies have been humanized to reduce immunogenicity, antibodies against these treatments can still form. All studies assessed for the presence of anti-drug antibodies, which were sometimes present before treatment. Anti-drug antibodies did not appear to affect drug concentration, efficacy, or adverse events.

All of the phase II trials administered treatment for 3 months with the exception of eptinezumab, which administered a single monthly dose. Thus, long-term safety is entirely unknown at this time. Open-label treatment phases lasting 1 year or longer will provide essential, longer-term safety and tolerability data.

The full range of CGRP’s physiologic functions is complex [43]. CGRP is a potent vasodilator, and thus, a theoretical risk exists that CGRP blockade could hinder vasodilation in physiologically appropriate situations such as cardiac or cerebrovascular ischemia. Indeed, animal data suggests that CGRP plays a protective role against cardiac ischemia, cerebrovascular ischemia, and reperfusion injury and vasospasm after subarachnoid hemorrhage [44•]. Additionally, since antibodies have a relatively long half-life, any untoward effects could not be quickly reversed.

Although no cardiovascular effects have been seen to date, the incidence of cardiovascular or cerebrovascular disease is very low in the populations studied; the mean age in the various trials ranged from 39 to 43 years. Much larger populations would be needed to see the effect of CGRP blockade on very rare events, and this would likely only be achieved with post-marketing surveillance. Furthermore, the potential for long-term effects of chronic CGRP inhibition over years even without overt ischemia is entirely unknown.

Lastly, CGRP receptors are found outside of the nervous and vascular systems, including in the adrenal glands, kidneys, pancreas, and bone. The effect of chronic CGRP antagonism on other organs is unknown.

## Discussion

Antagonists of the CGRP pathway comprise the first class of therapeutics targeted at a migraine-specific mechanism, and the use of monoclonal antibodies is a novel approach in migraine prevention. All four monoclonal antibodies in development have had positive phase II trials. In addition, it appears that efficacy might be seen more quickly with these antibodies than with current preventive options, which typically require up to 8 weeks of treatment, although this must be interpreted with caution. As prespecified additional endpoints, the primary outcome variable was also measured after the first and second treatment cycles (at weeks 4 and 8) in both of the fremanezumab trials; a significantly greater reduction compared with placebo was seen at both time points. Likewise, the erenumab 70 mg group, the only dose significantly different from placebo at 12 weeks, also showed a significant reduction in migraine days compared to placebo at weeks 4 and 8. A single dose of eptinezumab resulted in a significant reduction in migraine days in weeks 5 to 8, albeit with a one-side *p* value. A *post hoc* analysis conducted for the fremanezumab chronic migraine trial showed a significant reduction in number of headache hours compared to placebo within the first week of treatment [45].

Monoclonal antibodies are large molecules that cross the blood-brain barrier in a small ratio of 1:1000 [46], although in individual patients, the ratio may favor penetration more [47]. Thus, their site of action in migraine prevention is unclear. Additionally, while most acute treatments for migraine have the potential to worsen migraine with frequent use, antagonism of the CGRP pathway is effective both acutely and chronically for prevention. Thus, the efficacy of CGRP antagonists raises interesting questions about migraine pathophysiology and much remains to be understood.

The cost of treatment, once the monoclonal antibodies become commercially available, will certainly be high. In a healthcare system of limited resources, this cost will need to be balanced with the magnitude of benefit. The primary endpoint of migraine days is not a useful clinical measure since it presents the average of those that do well and those that do poorly. In line with this, trials also analyzed the 50, 75, or 100% responder rates, the proportion of patients achieving the respective reduction in the primary outcome, either as exploratory endpoints or in *post hoc* analyses. Most trials found significantly higher responder rates compared with placebo at week 12 and often earlier. An important task will be to attempt to identify patients more likely to benefit so personalized therapy can begin to offset the cost of the treatments by minimizing the number of patients treated who have no useful response.

An important group of patients, those that failed more than two preventive categories, was largely excluded from the trials, and thus, it is unknown what benefit this population would derive*.* While their exclusion from initial trials may be warranted to maximize sensitivity to detecting efficacy, from a clinical perspective, this group is of particular interest. Patients seen at specialty headache centers are often medically partially refractory, having failed a number of preventives with limited remaining options. This group potentially stands to benefit the most from a novel, migraine-specific mechanism.

While the long-term safety profile of the anti-CGRP antibodies remains to be seen, a well-tolerated treatment targeting a migraine-specific mechanism for the first time represents the most significant advance in migraine therapy in decades. A new era of migraine prevention is on the horizon, an exciting development for clinicians and patients alike.
